# Short-term fish predation destroys resilience of zooplankton communities and prevents recovery of phytoplankton control by zooplankton grazing

**DOI:** 10.1371/journal.pone.0212351

**Published:** 2019-02-15

**Authors:** Zeynep Ersoy, Sandra Brucet, Mireia Bartrons, Thomas Mehner

**Affiliations:** 1 Aquatic Ecology Group, University of Vic- Central University of Catalonia, Vic, Spain; 2 Department of Biology and Ecology of Fishes, Leibniz-Institute of Freshwater Ecology and Inland Fisheries (IGB), Berlin, Germany; 3 Catalan Institution for Research and Advanced Studies, ICREA, Barcelona, Spain; Swedish University of Agricultural Science, SWEDEN

## Abstract

Planktivorous fish predation directly affects zooplankton biomass, community and size structure, and may indirectly induce a trophic cascade to phytoplankton. However, it is not clear how quickly the zooplankton community structure and the cascading effects on phytoplankton recover to the unaffected state (i.e. resilience) once short-term predation by fish stops. The resilience has implications for the ecological quality and restoration measures in aquatic ecosystems. To assess the short-term zooplankton resilience against fish predation, we conducted a mesocosm experiment consisting of 10 enclosures, 6 with fish and 4 without fish. Plankton communities from a natural lake were used to establish phytoplankton and zooplankton in the mesocosms. High biomasses (about 20 g wet mass m^-3^) of juvenile planktivorous fish (perch, *Perca fluviatilis*) were allowed to feed on zooplankton in fish enclosures for four days. Thereafter, we removed fish and observed the recovery of the zooplankton community and its cascading effect on trophic interactions in comparison with no fish enclosures for four weeks. Short-term fish predation impaired resilience in zooplankton community by modifying community composition, as large zooplankton, such as calanoids, decreased just after fish predation and did not re-appear afterwards, whereas small cladocerans and rotifers proliferated. Total zooplankton biomass increased quickly within two weeks after fish removal, and at the end even exceeded the biomass measured before fish addition. Despite high biomass, the dominance of small zooplankton released phytoplankton from grazer control in fish enclosures. Accordingly, the zooplankton community did not recover from the effect of fish predation, indicating low short-term resilience. In contrast, in no fish enclosures without predation disturbance, a high zooplankton:phytoplankton biomass ratio accompanied by low phytoplankton yield (Chlorophyll-a:Total phosphorus ratio) reflected phytoplankton control by zooplankton over the experimental period. Comprehensive views on short and long-term resilience of zooplankton communities are essential for restoration and management strategies of aquatic ecosystems to better predict responses to global warming, such as higher densities of planktivorous fish.

## Introduction

Predators play a crucial role in food webs, by shaping the structure of prey communities and affecting ecosystem functioning, for example through trophic cascades, modification of energy flow and altered biodiversity [1–3]. In aquatic ecosystems, predation by planktivorous fish can strongly affect zooplankton biomass, community composition and size structure [4–7]. Several enclosure experiments and field studies have demonstrated that planktivorous fish predation caused a decrease in abundance of big cladocerans (e.g *Daphnia*) while favoring small cladocerans (e.g *Bosmina*, *Chydorus*), copepods and rotifers [8–10]. Fish predation can also have indirect effects on phytoplankton communities either through trophic cascades [11–13] or nutrient recycling [14–16]. Phytoplankton could benefit from controlled zooplankton grazing by fish and/or extra nutrients enhanced by fish resuspension [17] or excretion [18]. For this reason, lake restoration measures like biomanipulation mostly focus on reducing fish predation on zooplankton by planktivorous fish removal. This favors the recovery of large-sized zooplankton, which are the most efficient phytoplankton grazers, and leads to improvement of water quality [19–21].

With the increase of anthropogenic influences including climate change, habitat disturbance, overfishing and introduced species, the need to understand aquatic ecosystems’ resilience to disturbance has become more urgent. Resilience is defined as the ability of a system to recover after a disturbance and return to pre-disturbance state [22,23]. An example of disturbance for aquatic ecosystems could be higher densities of planktivorous fish, for example caused by climate warming [24,25] or fish stocking, whose higher predation may induce trophic cascades and impair ecosystem functioning in terms of biodiversity and energy flow [12,26,27]. In this sense, the capacity to identify the time for recovery and re-organisation of the zooplankton community structure after planktivorous fish removal is crucial for application of management strategies aimed to restore lake ecological status, as well as to understand failures in management [28–30].

Although there are many studies investigating the cascading effects of fish predation on zooplankton and phytoplankton communities [31–34], there are only a few focusing on zooplankton communities’ resilience to predation [30,35–38]. These former studies investigated the zooplankton communities in several lakes in North America, which had a history of fish stocking but experienced gradual fish removal or disappearance due to unsuitable spawning grounds. Subsequently, the zooplankton community returned within a few years to their previous conditions characterized by large-sized taxa, indicating long-term resilience. However, it remains unanswered how fast a zooplankton community recovers, once fish predation is completely stopped, in the temporal dimension of days or weeks. Answering this question could potentially improve our understanding about short-term resilience and stability of zooplankton communities and may help developing better management and conservation measures after sudden changes in freshwater ecosystems.

Here, we tested the short-term resilience of the zooplankton community to fish predation using a mesocosm experiment. We further assessed whether the potential recovery of zooplankton biomass after the stop of fish predation induced a comparably quick recovery of the top-down control by zooplankton on phytoplankton (expressed as zooplankton:phytoplankton biomass (zoo:phyto biomass) and chlorophyll-a:total phosphorus ratios (chla:TP)). We hypothesized that size-selective fish predation would affect the zooplankton biomass and community composition and would shift mean length towards smaller individuals [5,26], hence reducing top-down control on phytoplankton and increasing phytoplankton yield (chla:TP) [26,39]. We further expected that the zooplankton community is highly resilient and hence would quickly return to the pre-disturbance attributes within a few days after stop of predation. However, cascading effects on phytoplankton were expected to show a time lag in the response because phytoplankton has short turnover rates and hence may profit from the temporally reduced zooplankton grazing.

## Materials and methods

### Ethics statement

The specific experiment was not seperately approved by an animal research ethics committe. However, there is an ethics approval for experimental work with perch, issued to TM (Ernährung / Verhaltenstypen / Fische–G 0115 / 14, Landesamt für Gesundheit und Soziales Berlin, Germany). Animal procedures were conducted following German Animal Welfare Laboratory Regulations (Tierschutzversuchstier-verordnung, Anlage 2 TierSchVersV, https://www.gesetze-im-internet.de/tierschversv/BJNR312600013.html). Our study did not involve endangered or protected species. Fish were euthanized with 9:1 95% EtOH:clove-oil solution (CarlRoth, Karlsruhe, Germany) and a subsequent hit on the head.

The experiments were conducted at the ground of the Leibniz Institute of Freshwater Ecology and Inland Fisheries in Berlin, and hence no specific permission to conduct this study was needed.

### Experimental setup

**Fig 1:**
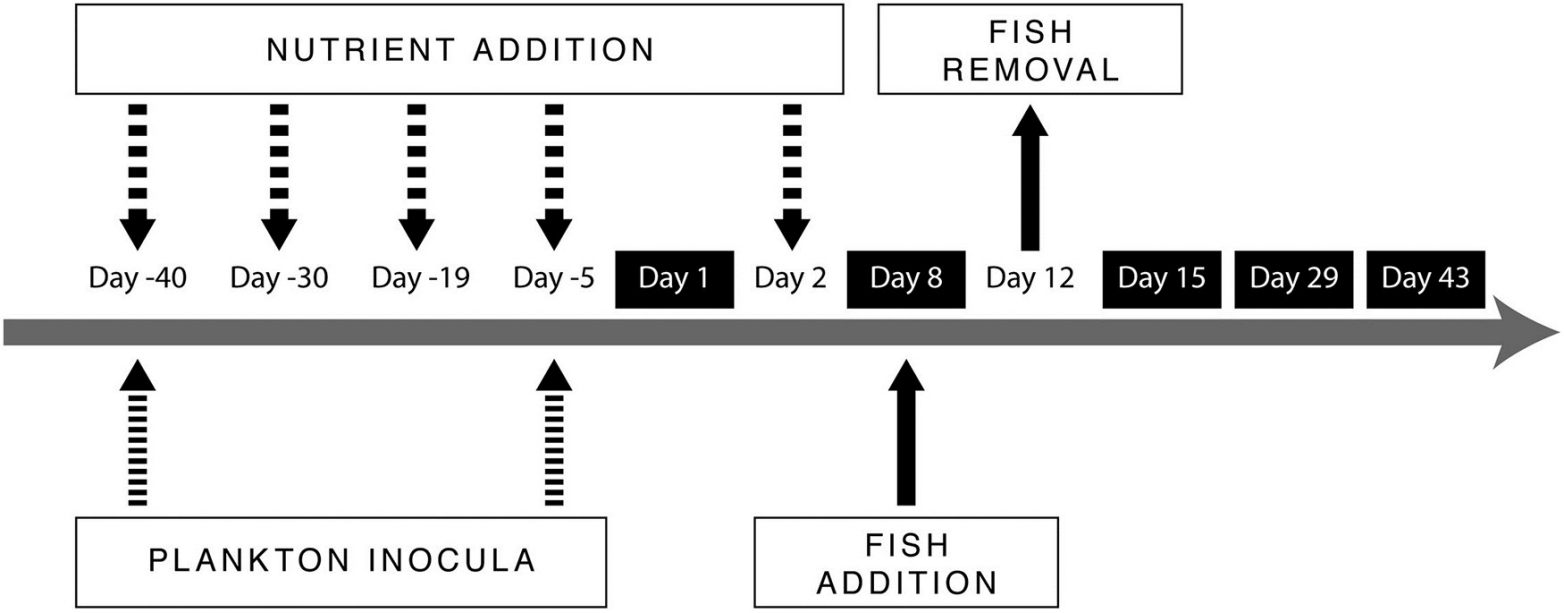
Experimental schedule showing plankton inoculation, fish addition, removal and sampling days. Days enframed with black show the sampling days. Day numbers with negative sign indicate the experimental manipulations before the experiment starts.

We established 12 circular and closed enclosures (diameter: 1.2 m) inside a small channel connected to Lake Müggelsee at the IGB’s ground in Berlin (52°26'53.1"N, 13°38'52.6"E) (ca. 80–90 cm deep). The initial water level in all enclosures was 1 m (~1000 L) and this did not change substantially during the experiment. To avoid stratification and ensure homogeneity and mixing in the enclosures, small aquarium water pumps (Sera pond precision, pond pump SP 500, Heinsberg, Germany) were installed at the mid-bottom of each enclosure. Nets (5 x 5 cm) were placed above the enclosures to avoid impact from birds, falling leaves etc. Before the experiment started, plankton inoculum and nutrients were added on certain days (see day numbers with negative sign in Fig 1). The experiment lasted for 43 days, from 30 May to 11 July 2016 with five samplings (days 1, 8, 15, 29, 43).

### Nutrient addition

Both N and P were added before the experiment started to counteract the concentration decrease from denitrification and sedimentation and to enable appropriate plankton development throughout the experiment.

Na_2_HPO_4_ (Sodium phosphate dibasic dehydrate) and Ca(NO_3_)_2_ (Calcium nitrate tetrahydrate) were used as a P and N-source, respectively. The target nutrient concentrations were 175 μg P L^-1^ and 1.5 mg N L^-1^ in each enclosure, and hence initially 180 mg P and 1545 mg of N were added per enclosure. In response to declining nutrient concentrations as measured in the enclosures, nutrients were added at days -40 (i.e. 40 days before the first sampling), -30, -19, -5 and +2 to facilitate phytoplankton growth (Fig 1).

### Plankton inoculum

Zooplankton and phytoplankton inocula from Lake Müggelsee were used to establish plankton communities in the mesocosms. Before the experiment started, lake water (2000 L) was filtered through 30 μm mesh size and mixed to create a natural mix of phytoplankton and zooplankton (day -40). From the plankton mixture, 3.5 L was added to each enclosure. The water temperatures were low in spring 2016, and hence the zooplankton communities in Lake Müggelsee consisted of only few larger crustaceans. Therefore, a second inoculum of natural zooplankton was prepared at day -5 by filtering nearly 2300 L of water from the lake through 100 μm mesh and adding the content of two horizontal net hauls (mesh size: 100 μm, about 5 minutes duration). The zooplankton inoculum was gently mixed, and 1.5 L of the mixture was added to each mesocosm.

### Sampling and laboratory analysis

Sampling started at day 1 (Fig 1), about one week after the addition of nutrients and the second plankton inoculum which were conducted on day -5. Three water samples (about 7 L each) were taken with a depth-integrating tube sampler (length: about 50 cm) at the surface, from the middle and the bottom layer of each enclosure (depth: around 1 m) and mixed thoroughly for analysis of chemical and biological variables. This repeated sampling of the homogeneously mixed volume was needed to ensure sufficient numbers of all organisms for reliable abundance estimates. One part of mixed samples (about 1.5 L) was analysed for total phosphorus (TP, μg L^-1^) and chlorophyll-a (chla, μg L^-1^). TP was determined using ascorbic acid-molybdate complex following persulfate digestion [40]. For chla analyses, water samples (100–200 ml) were filtered through 25 mm diameter Sartorius MGF Glass-Microfiber Disc. The filters were placed into 2 ml reaction vessels, frozen at -80 °C, freeze dried and thereafter stored at -25 °C in the dark until analysis. Chla was measured using high performance liquid chromatography (HPLC) following methodology from Shatwell et al. [41]. For calculating phytoplankton biomass, we converted chla to dry weight biomass (μg L^-1^) by multiplying with 66 [42].

From the mixed water sample, another 5 L were filtered through a 30 μm mesh and stored in 4% formaldehyde solution for zooplankton quantification. Large zooplankton (cladocerans and copepods) were counted and their length measured under a stereomicroscope while rotifers and copepod nauplii were counted and measured under a light microscope. All organisms were identified to species level except some rotifers that were identified to genus level. We measured at least 20 individuals (if possible) from each taxon and counted at least 100 individuals of the most abundant taxa. We classified copepods as adults, copepodites and nauplii to account for differing abundances during ontogeny. For all zooplankton groups, we calculated biomass by using available allometric relationships between weight and body length [43–45].

### Fish addition and removal

**Fig 2:**
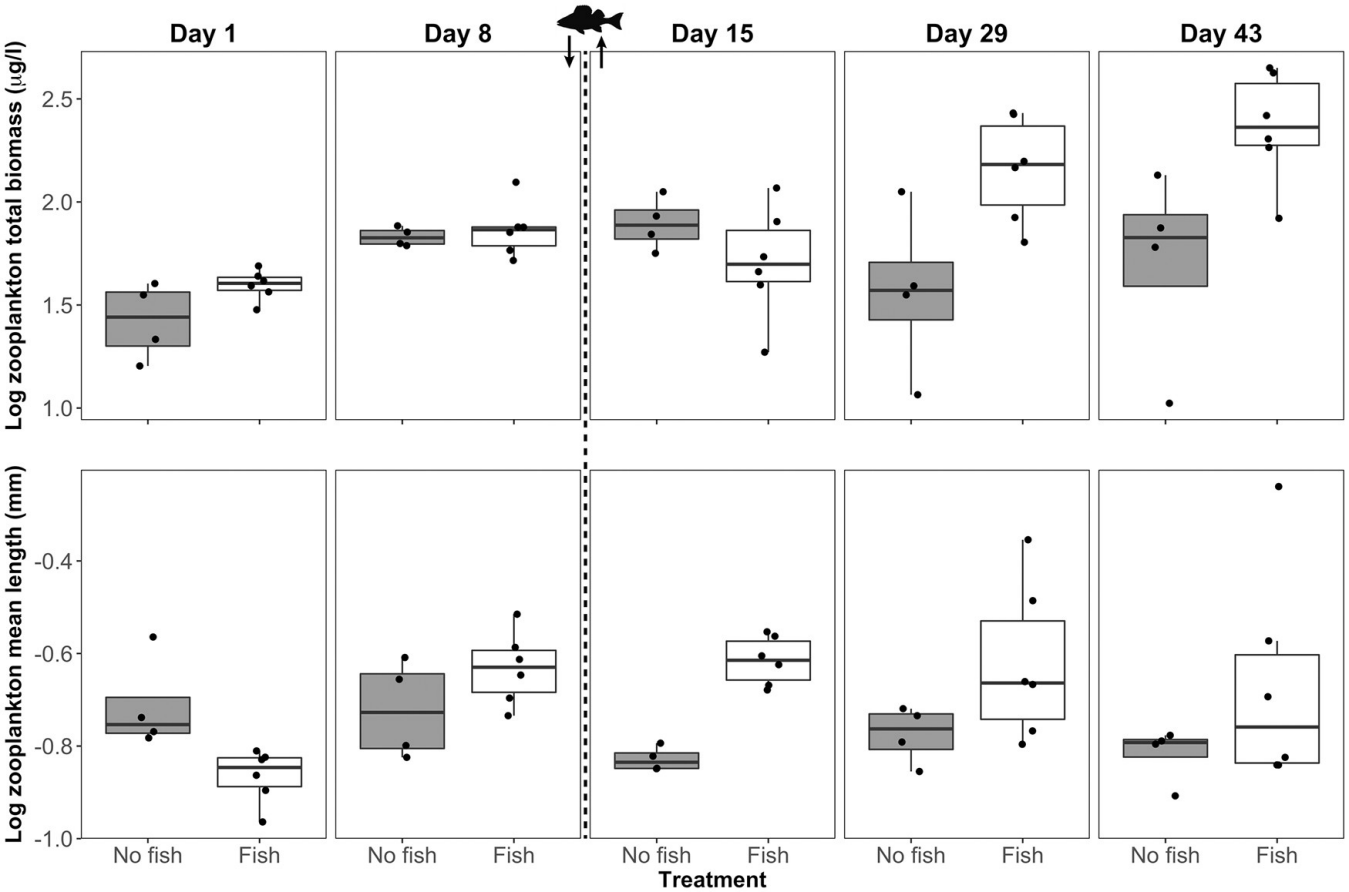
Log_10_ (zooplankton total biomass) and log_10_ (zooplankton mean length) for different treatments (no fish, n = 4; fish, n = 6) on each sampling day. Fish image with arrows indicate addition and removal of fish.

Juvenile European perch (*Perca fluviatilis*), which are typically planktivorous (Persson, 1990), were used as predators in the fish enclosures. Two to four weeks before the experiment, fish were caught by traps at the shoreline of Lake Müggelsee, held in aquaria with a continuous flow of filtered and oxygenated lake water from Lake Müggelsee. Therefore, the temperature in aquaria was similar to temperature in the lake at about 2 m depth. Fish were fed with *Tubifex* worms regularly. Fish were not fed during the two days before adding them to the enclosures to ensure that they were hungry enough to feed intensely on the zooplankton in the enclosures. Five perch of about 5 cm length and 4 g wet weight each were added to each fish enclosure (day 8). Fish were allowed to feed on zooplankton for four days. Average daily food consumption of a juvenile perch of 2–4 g is known to be around 4.5% of its biomass per day [46]. We estimated the daily food consumption by five perch (20 g) in one enclosure (~1000 L) to be about 900 μg L^-1^ day^-1^ (20 mg L^−1^ × 4.5%). Therefore, the daily consumption of all fish was substantially higher than the initially available zooplankton biomass in fish enclosures (around 500 μg L^-1^ at day 1, see values in Fig 2), indicating that strong predation effects were likely during four days of predation. Four days after fish stocking (day 12), we removed the fish by electrofishing, euthanized them with 9:1 95% EtOH:clove-oil solution (CarlRoth, Karlsruhe, Germany) and a subsequent hit on the head. We sampled the zooplankton and phytoplankton communities in both fish and no fish enclosures three times within the subsequent four weeks (days 15, 29, 43).

### Data analyses

**Fig 3:**
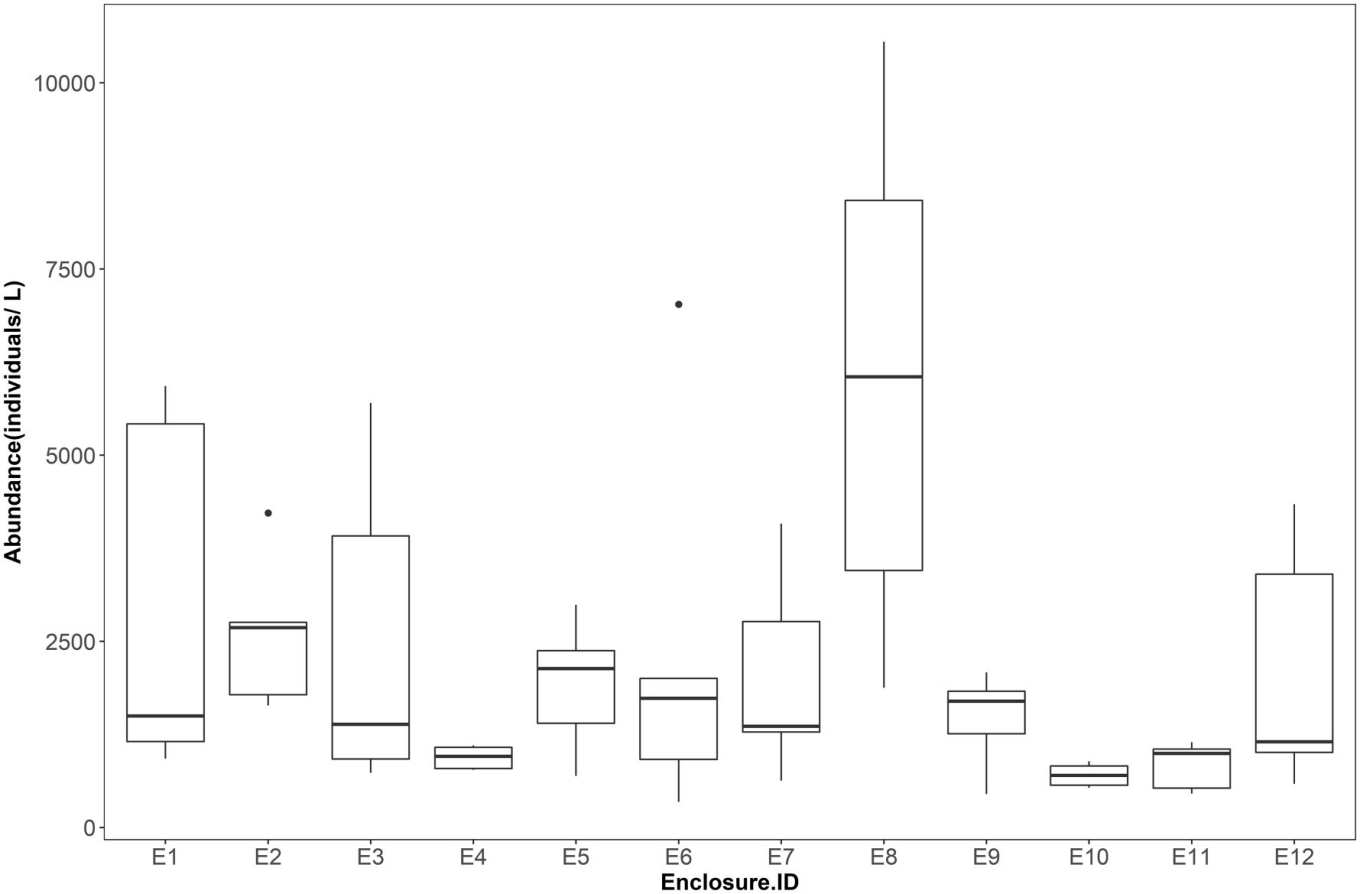
Total abundance of zooplankton in each enclosure. E1-8 are fish enclosures and E9-12 are no fish enclosures. Enclosures E5 and E8 were excluded because there was dead fish in E5 because of pump malfunctioning, and enclosure E8 stocked with fish became an extreme outlier in terms of total zooplankton abundance because of a massive rotifer bloom.

We excluded two fish enclosures from the data analyses because throughout the experiment, there were dead fish in one enclosure because of pump malfunctioning, and one enclosure stocked with fish became an extreme outlier in terms of total zooplankton abundance because of a massive rotifer bloom (See E8 in Fig 3). Finally, we used four enclosures without fish and six enclosures stocked with fish in our data analyses. Moreover, we assumed that the chla:TP ratio measured on day 1 was similar to that on day 8 (both dates before fish stocking), because we did not measure chla and TP on day 8.

We calculated linear mixed models (LMMs) to assess changes in predation pressure on zooplankton and cascading effects on phytoplankton communities between fish and no fish enclosures during the experiment. These LMMs included all sampling days during the experiment, and address the response of the variables to fish stocking combined with the temporal development of the variables in the enclosures. We tested the following variables of zooplankton community and size structure: 1) zooplankton total biomass, 2) zooplankton mean length, 3) zooplankton:phytoplankton biomass ratio as an indicator of top-down control on zooplankton [6,26,47,48] and 4) chla:TP ratio as an indicator of phytoplankton yield [6,49]. Firstly, we checked for temporal correlation by adding first order autocorrelation structure (correlation = corCAR1(form = ~Sampling Day|Enclosure ID)) [50] on the random-effects variance-covariance matrix of latent variables of the LMMs in “nlme” package [51]. Then, we compared the models (fitted with maximum likelihood estimation) with and without autocorrelation structure using likelihood ratio tests. There were no significant differences between these models (p>0.05), and hence we removed the temporal autocorrelation structure from the models. Accordingly, we used fish treatment and sampling day as fixed factors, and enclosure ID was modeled as a random factor. We checked the diagnostic plots of residuals of the models for the homogeneity of variance and tested the normality of residuals by Shapiro–Wilk’s test (p > 0.05). Variables were log transformed to achieve normal distributions and match the requirements of the statistical test. A significant interaction between treatment (fish, no fish) and sampling day would indicate that stocking and removal of fish in the fish enclosures modified zooplankton community and strength of trophic interactions over time differently in the fish than in the no fish enclosures.

Additionally, we tested for the resilience of the plankton communities by comparing zooplankton biomass, zooplankton mean size, zooplankton:phytoplankton biomass and chla:TP ratios between the sampling day immediately before fish stocking (day 8) and at the end of the experiment (day 43), separately for fish and no fish enclosures. The plankton communities would be considered resilient if there were no differences in these variables between the two sampling days, indicating that the plankton communities have returned to their pre-disturbance state within five weeks. These planned contrasts were estimated by paired Student’s and Welch’s t-tests according to checks for normality with Shapiro–Wilk’s test (p > 0.05) and the homogeneity of variances with F-tests. We corrected for multiple comparisons for each response variable for fish and no fish enclosures using Bonferroni method to avoid Type 1 error (adjusted p-value = α / number of tests). Then, the results of planned contrasts were considered significant for p<0.025 (α = 0.05).

All analyses were performed using “nlme” package [51] and all graphs were plotted using “ggplot2” package [52] in R version 3.4.3 [53].

## Results

The interaction between fish treatment and sampling day in the LMMs was significant for zooplankton biomass, zooplankton mean length, zooplankton:phytoplankton ratio and chla:TP ratio (Table 1). These results suggest differing successions of the zooplankton and phytoplankton communities between fish and no fish enclosures. Because of the significant interactions between the main effects, we do not focus further on the main effects in isolation.

**Table 1:** Results of linear mixed models to test for differences in zooplankton total biomass, zooplankton mean length, zooplankton:phytoplankton biomass ratio and chla:TP ratio.

Response variable	Predictors	df	F value	p-value
log_10_(zooplankton total biomass)	Fish	1	12.5	**0.0077**
	Sampling day	4	7	**0.0004**
	Fish*Sampling day	4	5.11	**0.0027**
log_10_(zooplankton mean length)	Fish	1	3.46	0.1
	Sampling day	4	2.68	**0.0494**
	Fish*Sampling day	4	4.49	**0.0054**
log_10_ (zoo:phyto biomass)	Fish	1	3.55	0.0961
	Sampling day	4	6.89	**0.0004**
	Fish*Sampling day	4	2.71	**0.0473**
log_10_ (chla:TP)	Fish	1	13.64	**0.0061**
	Sampling day	4	2.65	0.0509
	Fish*Sampling day	4	2.69	**0.0486**

Significant p-values are highlighted in bold.

**Fig 4:**
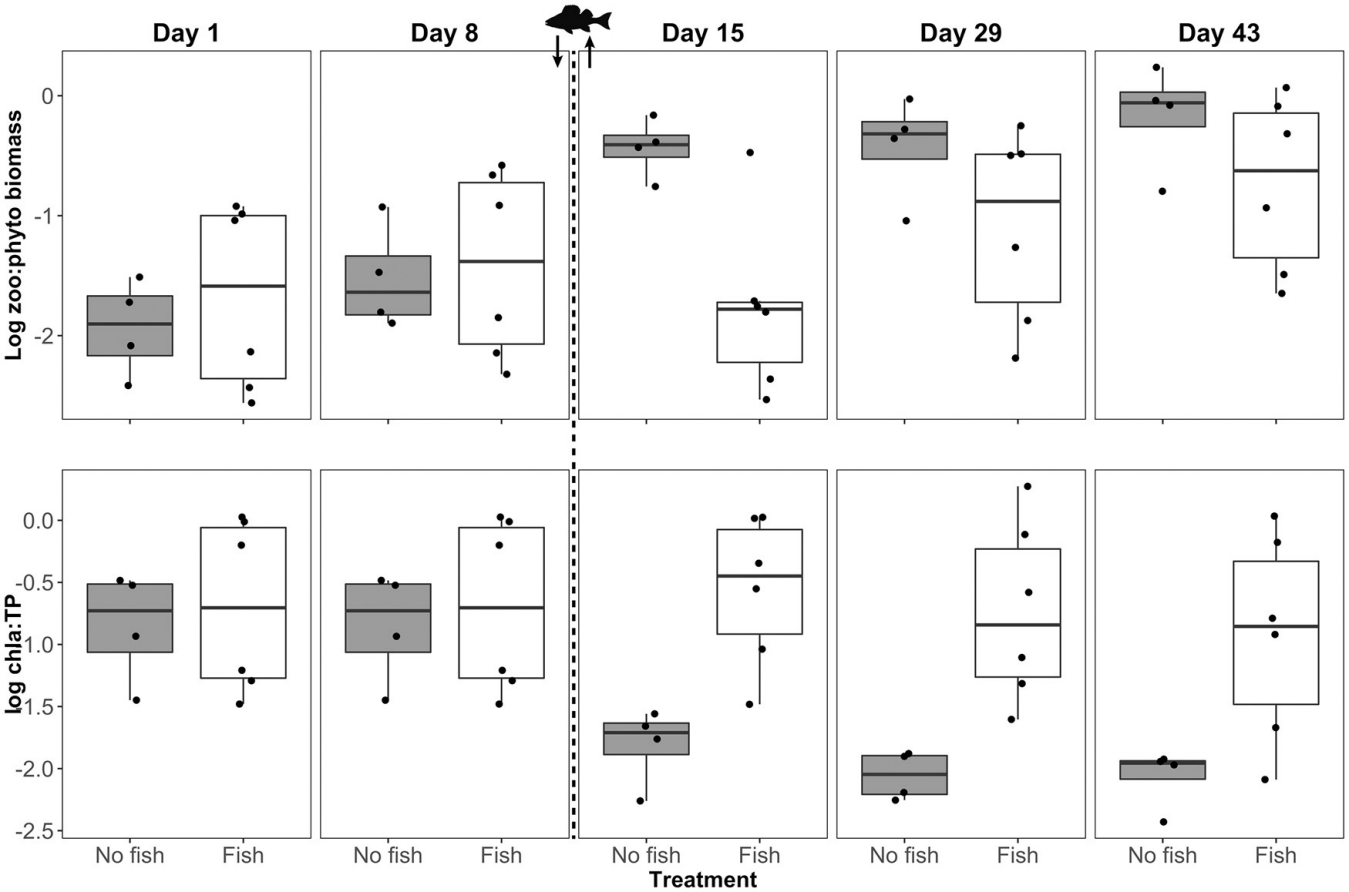
Log_10_(zooplankton:phytoplankton biomass) and log_10_(chla:TP) and ratio for different treatments (no fish, n = 4; fish, n = 6) on each sampling day. Fish image with arrows indicate addition and removal of fish.

To evaluate the resilience of the plankton communities, planned contrasts between day 8 (before fish stocking) and day 43 (end of experiment) revealed that zooplankton biomass was significantly higher in fish enclosures at day 43 than at day 8. The median of total zooplankton biomass across the six enclosures increased from 73 to 232 μg L^-1^. The median of zooplankton mean length across the six fish enclosures declined from 0.23 μm (day 8) to 0.18 μm (day 43) (Fig 2). However, zooplankton mean length was not significantly different between these days (Table 2), certainly caused by one strongly deviating enclosure in which the zooplankton length was high at day 43 (Fig 2). In the no fish enclosures, there were no differences in zooplankton biomass and mean length between days 8 and 43. In contrast, the zooplankton:phytoplankton biomass ratio was significantly higher at the end of the experiment compared to the sampling at day 8, whereas the chla:TP ratio was lower in the no fish enclosures (Fig 4). Both ratios did not differ between days 8 and 43 in the fish enclosures (Table 2).

**Table 2:** Results for contrasts between day 8 and day 43 for each response variable.

Response variable	Treatment	t value	p-value
log10(zooplankton total biomass)	Fish	-3.50	**0.017**
	No fish	0.50	0.65
log10(zooplankton mean length)	Fish	0.34	0.75
	No fish	2.16	0.12
log10 (zoo:phyto biomass)	Fish	-1.62	0.17
	No fish	-5.71	**0.01**
log10 (chla:TP)	Fish	0.55	0.60
	No fish	4.91	**0.016**

Significant p-values are highlighted in bold.

**Fig 5:**
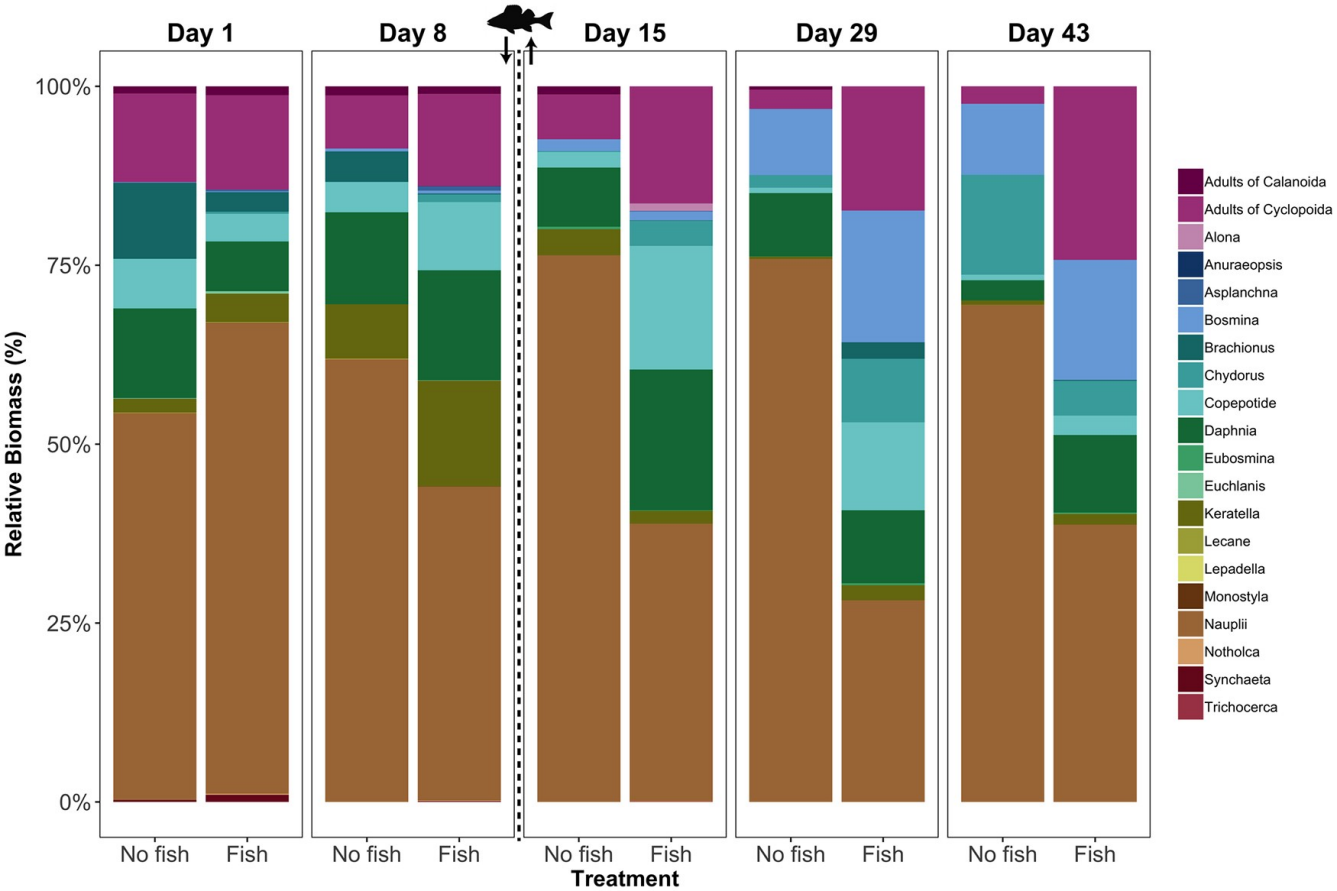
Relative biomass of different zooplankton taxa for different treatments (no fish, n = 4; fish, n = 6) on each sampling day. Fish image with arrows indicate addition and removal of fish.

The zooplankton community composition was modified in response to fish predation. Relative biomasses of nauplii and Calanoida adults declined immediately after fish predation in fish enclosures. Calanoida adults did not re-appear afterwards. Before the fish addition, *Daphnia* had higher biomass relative to other Cladocera taxa. However, after fish removal, Cladocera consisted mostly of small-sized taxa (*Bosmina* and *Chydorus*) and their relative contributions increased strongly during the last four weeks of the experiment in the fish enclosures compared to the period before fish addition (Fig 5). In contrast, the community composition in the no fish enclosures remained relatively stable (Fig 5).

## Discussion

Our results suggest that the predation on zooplankton by planktivorous fish in the few days between stocking and removal (i.e. short-term disturbance) caused significant changes of the zooplankton community in the fish relative to the no fish enclosures. Zooplankton biomass was higher at the end of the experiment than before fish predation in fish enclosures, whereas it did not change substantially in the no fish enclosures without disturbance. The indicators of trophic interactions (zooplankton:phytoplankton biomass and chla:TP ratios) reflected control of phytoplankton by grazing zooplankton only in the no fish enclosures, where high grazing pressure (i.e. high zoo:phyto biomass) decreased phytoplankton yield (i.e. low chla:TP). In contrast, phytoplankton was not under effective grazer control in the fish enclosures even after the stop of fish predation. Presumably, the shift in zooplankton community and size structure in response to fish predation prevented a trophic cascade down to phytoplankton, and hence phytoplankton proliferated despite high zooplankton biomasses in the fish enclosures. These effects were not transient, but persisted until the end of the experiment, indicating that the zooplankton communities were not resilient to strong, albeit short, fish predation effects.

Zooplankton biomass increased after stop of fish predation in fish enclosures, even reaching higher biomasses at the end than before fish addition. Furthermore, there was a slight trend that zooplankton mean length declined during this period in the fish enclosures. These results partly contrast with the results of other studies where fish predation caused a decline in biomass and mean length of the zooplankton community, because large-sized individuals decreased and small-sized ones dominated [5,11,54,55]. However, the earlier studies reflect the effect of permanent fish predation. In contrast, we evaluated the changes in the zooplankton community immediately after the stop of fish predation. In this sense, we focus on the effect of a short-term disturbance on zooplankton-phytoplankton interactions, in comparison with otherwise similar, but non-disturbed systems.

Obviously, fish predation, which lasted four days only, has changed the zooplankton community composition more than the zooplankton biomass in the fish enclosures, as shown by the development of different zooplankton taxa after fish removal. The biomass of large-sized taxa such as calanoids declined after fish stocking and did not increase again during the experiment, even when fish were removed. This result is consistent with previous similar studies [38,56], where the large calanoid copepod *Hesperodiaptomus* did not re-appear for several years even after fish disappearance. Together with their relatively low abundance, this delay of recovery may be attributed to the low metabolic rates and complex reproduction strategies of calanoid copepods [57]. Because copepods are obligate dioecious, mate limitation could decrease biomass and delay improvement in sexually reproducing populations [58,59]. Surprisingly, the biomass of similarly sized big Cladocera like *Daphnia* was less affected by predation, but the contribution of *Daphnia* to total zooplankton biomass was minor at the end of the experiment because the number of relatively small Cladocera taxa (e.g *Chydorus*, *Bosmina*) increased rapidly. These small taxa may have profited from the warm temperatures (between 20°C and 22°C from end of May until mid-July) and a quick maturation from their juvenile stages [60,61]. Accordingly, the disturbance by fish predation provided a ‘window of opportunity’ for the small cladocerans, and hence the zooplankton community composition did not recover to the original state from before the disturbance. The changes in community composition in the fish enclosures observed between days 8 and 43 of our experiment cannot be attributed to seasonal effects, since zooplankton biomass and mean length were relatively constant in the no fish enclosures without disturbance.

Interestingly, the shifts in zooplankton community composition as induced by fish predation prevented an effective phytoplankton control even after the fish predation has stopped. It has been shown several times that small zooplankton taxa are less efficient than large species to suppress phytoplankton biomass, even if they occur in high biomasses [62–64]. Accordingly, we observed both a high zooplankton:phytoplankton biomass ratio and a high chla:TP ratio in the fish enclosures, indicating high phytoplankton biomasses and yield at high zooplankton biomasses. In contrast, the zooplankton community in the no fish enclosures remained relatively stable, but the control of phytoplankton by zooplankton grazers became stronger towards the end of the experiment, indicated by a high zooplankton:phytoplankton biomass ratio, but a low phytoplankton yield. Therefore, it is surprising to see that the enclosures strongly differed at the end of the experiment with respect to the strength of the zooplankton-phytoplankton interaction, although all enclosures had no fish at this time, and zooplankton was exposed to fish predation only for four out of 43 days in the fish enclosures. Therefore, the legacy of short-term predation and disturbance had long-lasting effects on trophic interactions, reflecting weak short-term resilience of zooplankton to fish predation. Monitoring studies in lakes suggest, however, that larger zooplankton species may recover after stop of fish predation in the long-term, suggesting that only short-term resilience of zooplankton may be impaired by massive disturbance [65,66]. Seasonality and other environmental factors can also influence these resilience mechanisms by modifying population dynamics [67].

We recognize that our experimental design had certain limitations. Although mesocosms are helpful for mechanistic studies, their use has limitations when complex interactions and long-term responses have to be explored [68,69]. Moreover, in our experimental set-up we considered a simple three trophic level cascade and ignored the effects of omnivory, intraguild predation, ontogenetic changes, the contribution of the microbial loop to food web interactions [70–72] and the role of resting stage banks in natural communities [35]. Recolonization of zooplankton after the removal of a predation disturbance could be promoted by emergence from the resting eggs from the sediment [73] or dispersal from nearby aquatic ecosystems by means of water birds, amphipods or semi-aquatic mammals [74–76]. Moreover, in natural ecosystems, diel horizontal (to plants) or vertical (to bottom) migration of zooplankton could enhance the resistance of zooplankton to fish predation [77,78]. However, these defense mechanisms depend highly on the climate and physical characteristics of the ecosystems such as temperature and turbidity [54,79,80]. Therefore, it is crucial to consider these site and climate specific factors in natural ecosystems before applying restoration measures.

A recent study investigating multiple dimensions of stability of freshwater ecosystems to single perturbations has found that the recovery in the ecosystem functioning was highly related to the recovery in the community composition of plankton in mesocosms [81]. Within this context, our findings could be relevant for better restoration and management strategies in a rapidly changing world. Increased climate warming and invasive species could exacerbate resilience in large-sized zooplankton, which could have severe consequences for restoration measures [82,83]. Understanding and identifying the mechanisms of short-term and long-term resilience of natural communities will be essential for conserving the ecosystem functions and predict community dynamics in response to future disturbances [84].

## Supporting information

S1 Fig Log_10_ (TP) and log_10_ (chl-a) for different treatments (no fish, n = 4; fish, n = 6) on each sampling day. Fish image with arrows indicate addition and removal of fish.
(TIF)

S1 File (XLSX)
