# Correction: Short-term fish predation destroys resilience of zooplankton communities and prevents recovery of phytoplankton control by zooplankton grazing

**DOI:** 10.1371/journal.pone.0215078

**Published:** 2019-04-03

**Authors:** The PLOS ONE Staff

## Notice of Republication

An incorrect version of S1 File was published in error. The publisher apologizes for this error. This article was republished on March 27, 2019 to correct for this error. Please download this article again to view the correct version.

## References

[1] Ersoy Z, Brucet S, Bartrons M, Mehner T (2019) Short-term fish predation destroys resilience of zooplankton communities and prevents recovery of phytoplankton control by zooplankton grazing. PLoS ONE 14(2): e0212351 10.1371/journal.pone.0212351 30768619 PMC6377254

