# Supplementary figures and images for: Short-term fish predation destroys resilience of zooplankton communities and prevents recovery of phytoplankton control by zooplankton grazing
Source: PLoS One. 2019 Feb 15;14(2):e0212351. doi: 10.1371/journal.pone.0212351 (PMC6377254; doi:10.1371/journal.pone.0212351)

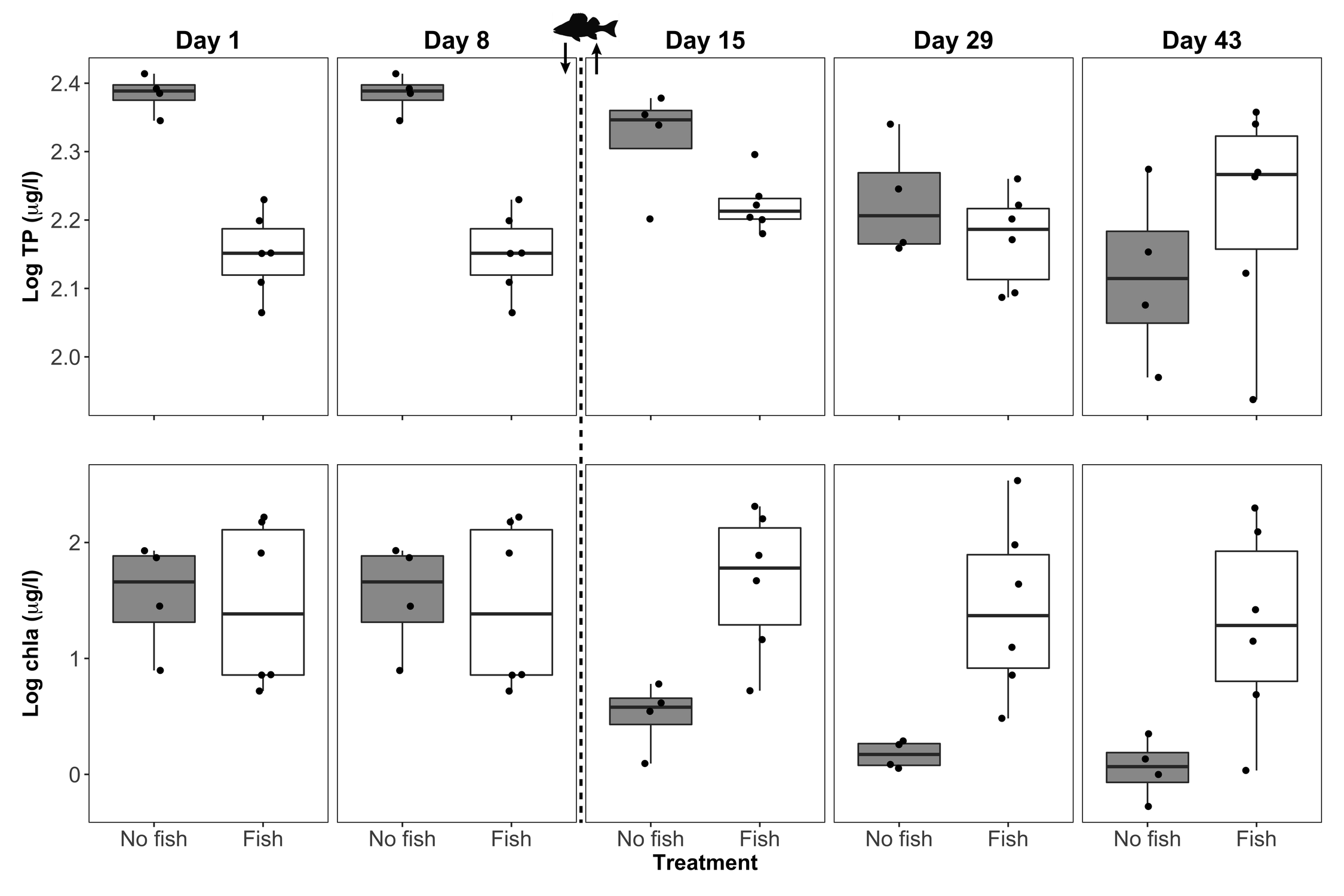

Supplement: S1 Fig — Fish image with arrows indicate addition and removal of fish. (TIF) [file pone.0212351.s001.tif]
